# Profiling steroid and thyroid hormones with hair analysis in a cohort of women aged 25 to 45 years old

**DOI:** 10.1530/EJE-22-0081

**Published:** 2022-02-21

**Authors:** Feng-Jiao Peng, Paul Palazzi, Sakina Mezzache, Nasrine Bourokba, Jeremie Soeur, Brice M R Appenzeller

**Affiliations:** 1 Human Biomonitoring Research Unit, Department of Precision Health, Luxembourg Institute of Health, Strassen, Luxembourg; 2 L’Oréal Research and Innovation, Aulnay sous Bois, France; 3 L’Oréal Research and Innovation, Synapse, Singapore

## Abstract

**Objective:**

Endogenous hormones regulate numerous physiological processes in humans. Some of them are routinely measured in blood, saliva and/or urine for the diagnosis of disorders. The analysis of fluids may, however, require multiple samples collected at different time points to avoid the high variability in the concentration of some hormones. In contrast, hair analysis has been proposed as an interesting alternative to reveal average hormone levels over a longer period. In this work, we developed and validated an analytical method for analyzing 36 endogenous steroid and thyroid hormones and one pineal hormone in human hair using ultra-performance liquid chromatography (UPLC)-tandem mass spectrometry (MS/MS).

**Methods:**

Sample preparation involved hair decontamination, pulverization, methanol extraction, and purification with C_18_-solid phase extraction. Extracts were then divided into two portions, respectively injected into an UPLC-MS/MS system, and analyzed using two different instrumental methods. The method was applied to a healthy female population aged 25–45 years.

**Results:**

The method was validated on supplemented hair samples for the 37 targeted hormones, and its application to the population under study allowed to detect 32 compounds in 2–100% of the samples. Complete reference intervals (2.5–97.5th percentiles) were established for estrone, 17β-estradiol, androstenedione, dehydroepiandrosterone, progesterone, 17α-hydroxyprogesterone, cortisone, cortisol and 3,3’,5-triiodo-L-thyronine. Hair cortisone, cortisol, tetrahydrocortisone and tetrahydrocortisol concentrations were highly correlated with each other, with Kendall’s τ correlation coefficients ranging from 0.52 to 0.68.

**Conclusion:**

Allowing the detection of 32 hormones from different chemical classes, the present method will allow to broaden hormonal profiling for better identifying endocrine disorders.

## Introduction

Endogenous hormones regulate numerous physiological processes in humans, such as sexual maturation, immune function, metabolism and neurodevelopment (1, 2, 3). Some steroid (e.g. cortisol (F), testosterone, 17β-estradiol (E2) and progesterone (P4)) and thyroid (e.g. 3,3’,5-triiodo-L-thyronine (T3) and 3,5,3’,5’-triiodo-L-thyronine (T4)) hormones are routinely measured in blood, saliva and/or urine samples for evaluation of hormonal homeostasis and diagnosis of disorders such as Cushing syndrome, polycystic ovary syndrome and hypothyroidism. Such measurements reflect either circulating hormone concentrations (blood or saliva) or their integration over hours (urine) (4). However, it is difficult to extrapolate long-term hormonal levels from these measures because hormonal secretion shows ultradian, circadian and/or infradian variations (5). Consequently, revealing hormonal status may require the analysis of multiple samples collected at different time points. Alternatively, measurements in the hair are assumed to reflect average hormone concentration in the body over months because compounds are continuously incorporated from blood to hair follicles during hair growth (6). In addition, hormones captured inside the hair are stable and hair sampling is easy, noninvasive and stress-free. Therefore, hair analysis is gaining attention in the assessment of endogenous steroid hormones (e.g. linking hair F to chronic stress) (7).

Immunoassay, gas chromatography-mass spectrometry (GC-MS), and liquid chromatography-mass spectrometry (LC-MS) are the most common techniques used for quantifying steroid and thyroid hormones in human hair (8, 9). The immunoassay methods are simple and sensitive, but they can quantify only one hormone at a time and may suffer from insufficient specificity and accuracy. In contrast, both GC-MS and LC-MS methods allow the simultaneous determination of multiple hormones and are highly sensitive and selective. Nevertheless, GC-MS methods tend to have lower sensitivity and sample throughput and require a larger hair amount and more intensive sample preparation compared to LC-MS methods (10). In recent years, LC-MS methods have thus been widely used in hair analysis for hormonal assessment in humans. However, previously reported analytical methods focused only on a few hormones belonging mainly to androgens and glucocorticoids, and/or were applied only to a limited number of hair samples (Supplementary Table 1, see section on supplementary materials given at the end of this article). However, simultaneous analysis of multiclass hormones would supply a more comprehensive picture of hormonal homeostasis.

**Figure 1:**
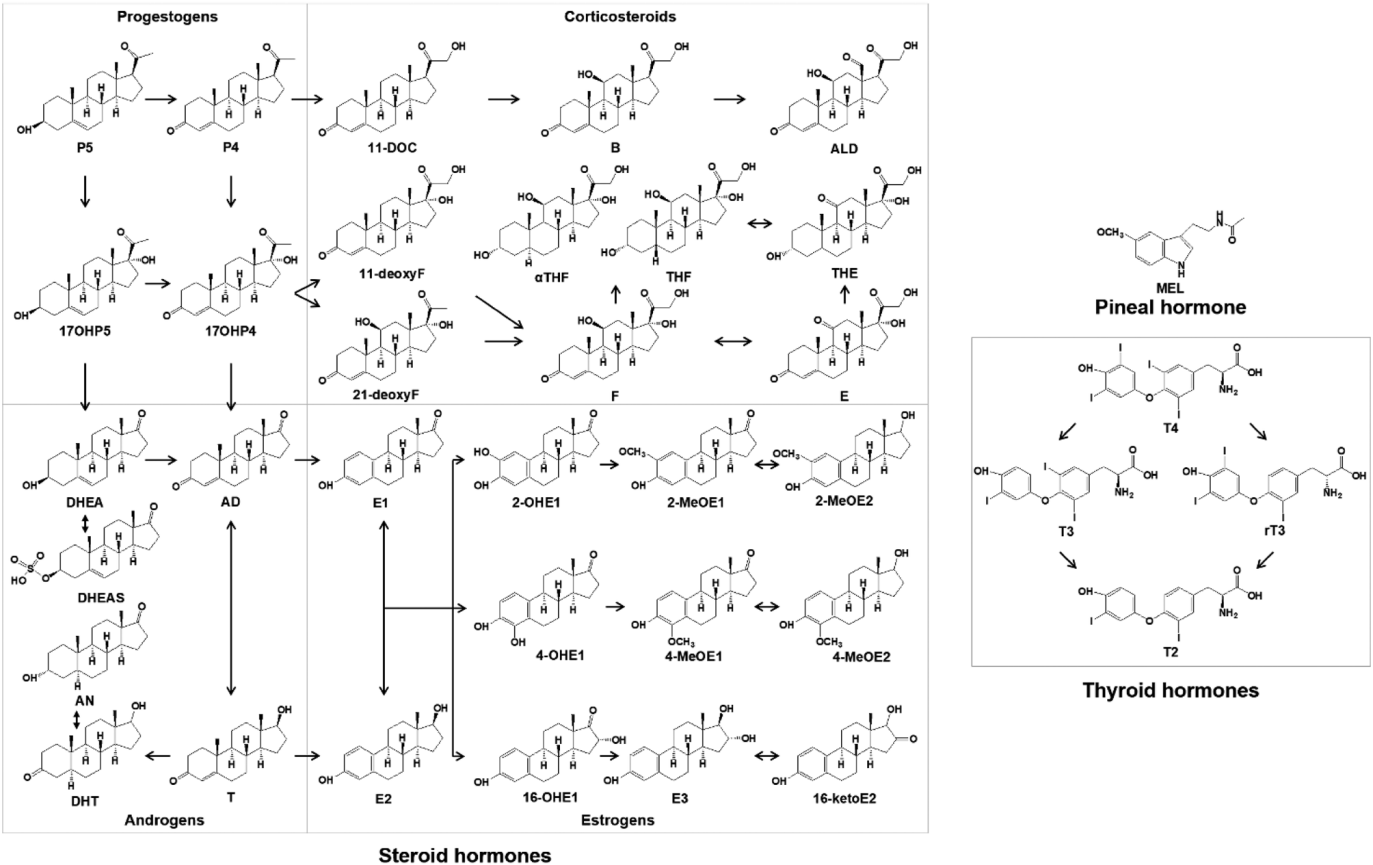
Biosynthesis pathways of hormones included in our method in the human body. Epitestosterone was not presented in this figure because it is not clear about its biological role and biosynthesis (28). AD, androstenedione; ALD, aldosterone; AN, androsterone; B, corticosterone; DHT, 5α-dihydrotestosterone; E, cortisone; E1, estrone; E2, 17β-estradiol; E3, estriol; F, cortisol; MEL, melatonin; P4, progesterone; P5, pregnenolone; rT3, 3,3’,5’-triiodo-L-thyronine; THE, tetrahydrocortisone; THF, tetrahydrocortisol; T2, 3,3’-diiodo-L-thyronine; T3, 3,3’,5-triiodo-L-thyronine; T4, 3,5,3’,5’-triiodo-L-thyronine; 2-MeOE1, 2-methoxyestrone; 2-MeOE2, 2-methoxyestradiol; 2-OHE1, 2-hydroxyestrone; 4-MeOE1, 4-methoxyestrone; 4-MeOE2, 4-methoxyestradiol; 4-OHE1, 4-hydroxyestrone; 11-deoxyF, 11-deoxycortisol; 11-DOC, 11-deoxycorticosterone; 16-ketoE2, 16-ketoestradiol; 16-OHE1, 16α-hydroxyestrone; 17OHP5, 17α-hydroxypregnenolone; 17OHP4, 17α-hydroxyprogesterone; 21-deoxyF, 21-deoxycortisol; αTHF, 5α-tetrahydrocortisol.

Here, we developed and validated an ultra-performance liquid chromatography (UPLC)-tandem mass spectrometry (MS/MS) assay for analyzing hair concentration of 37 endogenous hormones belonging to multiple classes, including steroid hormones, thyroid hormones and pineal hormones (Fig. 1). We also applied the developed method to hair samples collected from a cohort of healthy women aged 25–45 years.

## Subjects and methods

Targeted endogenous hormones consisted of 11 estrogens, 7 androgens, 4 progestogens, 10 corticosteroids, 4 thyroid hormones and 1 pineal hormone (Fig. 1). Hair samples were collected in 2016 from 196 healthy Chinese women aged between 25 and 45 years. The first 12-cm hair segment from the root was used to analyze hormones, which represents the average hormone levels over a 12-month period prior to hair sampling by assuming an average hair growth rate of 1 cm/month (11). The study was approved by the Ethics Committee of the Chinese Academy of Inspection and Quarantine Cosmetics Tech Center (protocol no. 2015-033-DY-024), and written consent has been obtained from each subject after a full explanation of the purpose and nature of all procedures used (12). Details on chemicals and reagents, preparation of stock and working standards, sample collection, sample preparation, LC-MS/MS method, method validation, and statistical analysis are described in the online Supplementary data file.

## Results

### Method validation

Results from the method validation are shown in Supplementary Table 2. For each analyte, the calibration curve showed adequate linearity with R^2^ ≥0.99. The lower limit of quantification (LLoQ) values ranged from 0.1 pg/mg for estrone (E1) and E2 to 10 pg/mg for androsterone (AN), pregnenolone (P5) and 17α-hydroxypregnenolone (17OHP5). Intra- and inter-assay accuracies and precisions were within the acceptable ranges. The mean recovery yielded from the three tested levels (1, 10, and 100 pg/mg) ranged from 57 to 103%. At the spiked concentration of 1 pg/mg, recovery was not determined for AN and 17OHP5 because of their low sensitivity. No significant interferences were observed for any analytes except 2-hydroxyestrone (2-OHE1) and 4-OHE1, which were thus quantified using the second most abundant product ion (m/z 235). Dehydroepiandrosterone sulfate (DHEAS) was quantified without confirmation transition due to the low ratio of the qualitative ion to the quantitative ion (0.007). For both standards and internal standards, no carry-over was detected.

### Hair hormones concentrations

Thirty-two out of the 37 target hormones were detected in at least one hair sample, and only 4-methoxyestrone (4-MeOE1), 16-ketoestradiol (16-ketoE2), AN, 17OHP5 and 21-deoxycortisol (21-deoxyF) were never detected (Table 1). At the 2.5th percentile, E1, E2, androstenedione (AD), DHEA, P4, 17α-hydroxyprogesterone (17OHP4), cortisone (E), F and T3 showed concentrations above their respective LLoQs. The reference interval (2.5–97.5th percentiles) for these nine hormones were 0.24–1.92, 0.19–0.95, 1.42–6.79, 4.04–19.8, 1.59–22.5, 1.07–4.92, 5.08–37.3, 1.91–11.0, and 0.32–0.76 pg/mg, respectively. In each hair sample, the number of detectable hormones ranged from 17 to 28, while the number of quantifiable hormones ranged from 10 to 20.

**Table 1:** Concentrations (pg/mg) of hormones measured in hair samples collected from 196 healthy women.

Hormone	DF (%)	Percentile					Range	LLoQ	LOD
		2.5th	25th	50th	75th	97.5th			
E1	99	0.24	0.72	0.91	1.22	1.92	nd–2.80	0.1	0.1
E2	100	0.19	0.28	0.39	0.53	0.95	0.07–1.71	0.1	0.07
E3	6	nd	nd	nd	nd	0.15	nd–1.14	0.5	0.09
2-OHE1	37	nd	nd	nd	0.43	1.50	nd–1.88	1	0.19
4-OHE1	2	nd	nd	nd	nd	nd	nd–8.90	2	0.42
16-OHE1	34	nd	nd	nd	0.09	0.31	nd–0.48	0.5	0.04
2-MeOE1	29	nd	nd	nd	0.15	0.87	nd–1.37	0.5	0.1
4-MeOE1	0	nd	nd	nd	nd	nd	nd	1	1
2+4-MeOE2	99	0.38	1.16	2.15	3.58	9.66	nd–13.3	0.5	0.15
16-ketoE2	0	nd	nd	nd	nd	nd	nd	1	1
AD	100	1.42	2.48	3.20	4.16	6.79	1.06–7.68	0.5	0.5
T	95	nd	0.32	0.42	0.58	0.96	nd–1.10	0.5	0.15
EpiT	33	nd	nd	nd	0.40	0.89	nd–1.82	0.5	0.22
DHT	62	nd	nd	0.77	1.62	6.24	nd–7.88	0.5	0.36
DHEA	100	4.04	7.05	8.88	11.3	19.8	0.72–25.6	2	0.72
DHEAS	100	0.71	1.53	2.27	3.71	17.4	0.44–20.7	5	0.44
AN	0	nd	nd	nd	nd	nd	nd	10	10
P5	58	nd	nd	8.01	15.2	34.4	nd–44.2	10	4.45
17OHP5	0	nd	nd	nd	nd	nd	nd	10	10
P4	100	1.59	6.20	8.99	11.8	22.5	0.51–35.0	0.2	0.2
17OHP4	100	1.07	1.73	2.34	2.89	4.92	0.60–6.37	0.2	0.2
11-DOC	87	nd	0.14	0.19	0.23	0.38	nd–0.69	0.2	0.08
B	54	nd	nd	0.61	1.01	2.17	nd–2.42	2	0.43
ALD	7	nd	nd	nd	nd	0.24	nd–0.47	2	0.09
11-deoxyF	19	nd	nd	nd	nd	0.28	nd–0.33	0.5	0.09
21-deoxyF	0	nd	nd	nd	nd	nd	nd	1	1
E	100	5.08	7.64	9.83	13.6	37.3	4.19–47.6	0.5	0.5
F	100	1.91	2.61	3.37	4.65	11.0	1.20–13.2	0.5	0.5
THE	100	0.50	0.80	1.07	1.40	3.36	0.38–5.51	1	0.38
THF	100	1.22	2.19	2.88	4.29	10.8	1.09–14.2	2	1.09
αTHF	73	nd	nd	1.23	2.08	4.76	nd–6.71	2	0.42
T2	68	nd	nd	0.17	0.24	0.42	nd–0.53	0.5	0.09
T3	100	0.32	0.43	0.51	0.58	0.76	0.29–1.18	0.2	0.2
rT3	93	nd	0.10	0.12	0.15	0.28	nd-0.51	0.5	0.07
T4	100	0.09	0.13	0.18	0.24	0.58	0.05–0.73	0.2	0.05
MEL	32	nd	nd	nd	0.09	2.89	nd–7.80	0.2	0.02

AD, androstenedione; AN, androsterone; ALD, aldosterone; B, corticosterone; DF, detection frequency; DHT, 5α-dihydrotestosterone; E, cortisone; EpiT, epitestosterone; E1, estrone; E2, 17β-estradiol; E3, estriol; F, cortisol; LOD, limit of detection; LLoQ, limit of quantification; MEL, melatonin; nd, not detected; P4, progesterone; P5, pregnenolone; THE, tetrahydrocortisone; THF, tetrahydrocortisol; T2, 3,3’-diiodo-L-thyronine; T3, 3,3’,5-triiodo-L-thyronine; rT3, 3,3’,5’-triiodo-L-thyronine; T4, 3,5,3’,5’-triiodo-L-thyronine; 2-MeOE1, 2-methoxyestrone; 2-OHE1, 2-hydroxyestrone; 4-MeOE1, 4-methoxyestrone; 4-OHE1, 4-hydroxyestrone; 2+4-MeOE2, 2-methoxyestradiol and 4-methoxyestradiol; 11-deoxyF, 11-deoxycortisol; 11-DOC, 11-deoxycorticosterone; 16-ketoE2, 16-ketoestradiol; 16-OHE1, 16α-hydroxyestrone; 17OHP4, 17α-hydroxyprogesterone; 17OHP5, 17α-hydroxypregnenolone; 21-deoxyF, 21-deoxycortisol; αTHF, 5α-tetrahydrocortisol.

### Correlations between hormones concentrations, age and BMI

**Figure 2:**
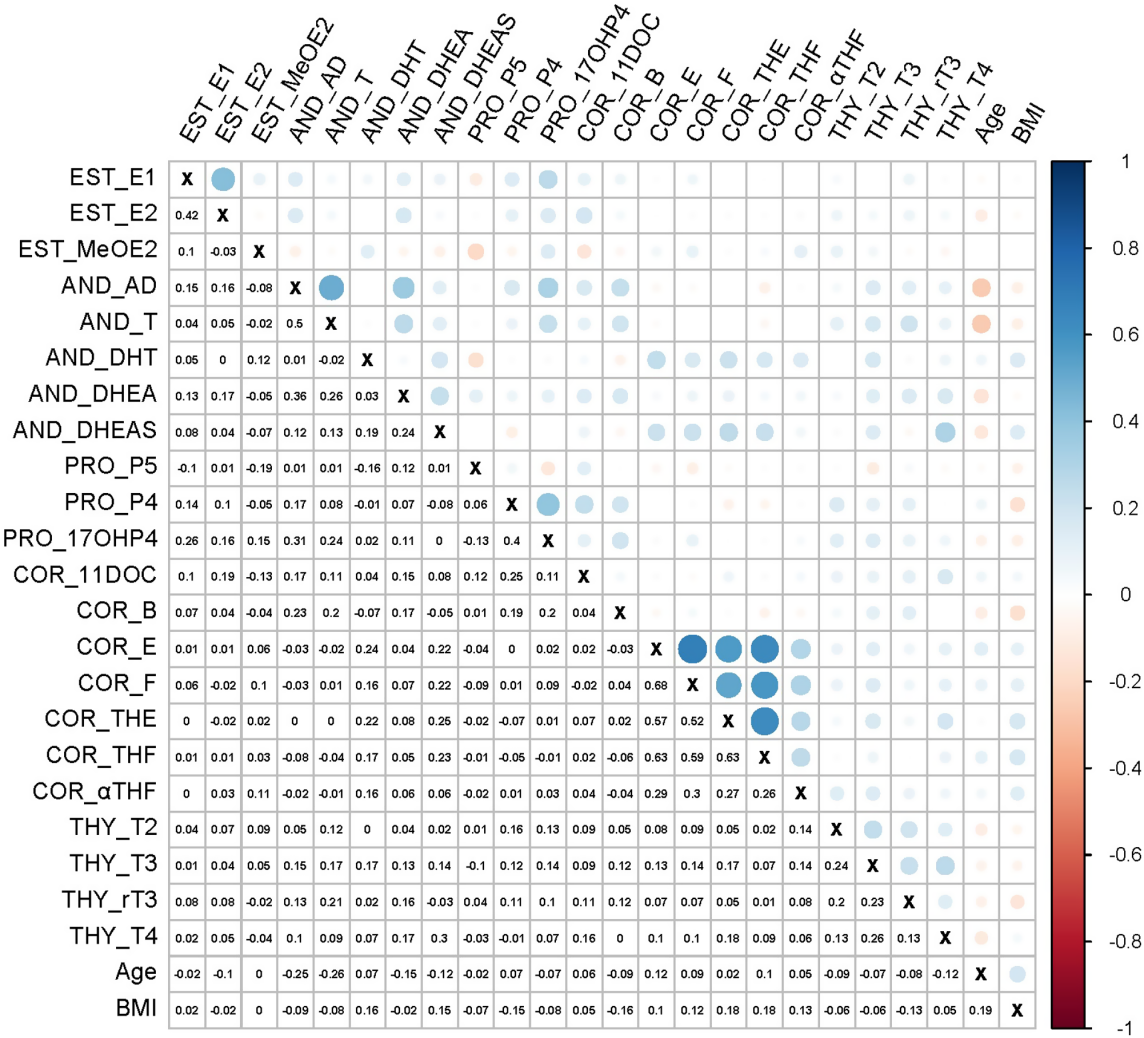
Heatmap showing pairwise Kendall correlations between analyte concentrations in hair samples collected from 196 healthy women. The size of circle symbols represents the strength of correlations. A cross symbolizes that the correlation coefficient is one. Only analytes detected in more than 50% of samples are presented in the graph. AD, androstenedione; AND, androgens; B, corticosterone; COR, corticosteroids; DHT, 5α-dihydrotestosterone; E, cortisone; EST, estrogens; E1, estrone; E2, 17β-estradiol; F, cortisol; MeOE2, 2-methoxyestradiol and 4-methoxyestradiol; PRO, progestogens; P4, progesterone; P5, pregnenolone; rT3, 3,3’,5’-triiodo-L-thyronine; THE, tetrahydrocortisone; THF, tetrahydrocortisol; THY, thyroid hormones; T2, 3,3’-diiodo-L-thyronine; T3, 3,3’,5-triiodo-L-thyronine; T4, 3,5,3’,5’-triiodo-L-thyronine; 11DOC, 11-deoxycorticosterone; 17OHP4, 17α-hydroxyprogesterone; αTHF, 5α-tetrahydrocortisol. A full color version of this figure is available at https://doi.org/10.1530/EJE-22-0081.

Significant correlations were observed between hormones, between hormones and age, and between hormones and BMI (Fig. 2). Specifically, the strongest correlations were found between four corticosteroids (i.e., E, F, tetrahydrocortisone (THE) and tetrahydrocortisol (THF); τ_Kendall_ = 0.52-0.68), followed by AD and T (τ_Kendall_ = 0.50), E1 and E2 (τ_Kendall_ = 0.42), P4 and 17OHP4 (τ_Kendall_ = 0.40), and AD and DHEA (τ_Kendall_ = 0.36) (Supplementary Fig. 1). Thyroid hormones were all significantly positively correlated with each other (τ_Kendall_ = 0.13-0.26; Supplementary Fig. 2). There were also significant positive correlations between hormones from different classes, for example, AD and 17OHP4, DHEAS and THE, and DHEAS and T4 (Supplementary Fig. 3). Women’s age was inversely correlated with AD, T, DHEA, DHEAS, T4 and 3,3’-diiodo-L-thyronine (T2) and positively with E, F, and THF (Supplementary Fig. 4). BMI was positively correlated with 5α-dihydrotestosterone, DHEAS, E, F, THE, THF, and αTHF but negatively correlated with P4 and 3,3’,5’-triiodo-L-thyronine (rT3) (Supplementary Fig. 5).

## Discussion

Previous studies on hormone analysis in hair have focused mostly on steroid hormones, especially T, E and F (Supplementary Table 1), though it has been well recognized that steroid hormones can interact with thyroid hormones in both humans and animals (13, 14). Simultaneously including estrogens, androgens, progestogens, corticosteroids, thyroid hormones and melatonin, the present method may broaden the information that can be obtained from hair analysis and provide a much more comprehensive picture of the hormonal status of the individual (Fig. 1). This method is the first to analyze aldosterone, T2, rT3 and melatonin in human hair. Although we used an amount of 50 mg per sample to take into account the low levels of some hormones in human hair, our method is also applicable to samples with a less amount (e.g. 30 mg), especially for hormones showing high levels (e.g. E, F, DHEA, and P4).

Applying the methodology to a healthy female population aged 25–45 years allowed to detect 32 hormones in at least one subject’s hair, and only 4-MeOE1, 16-ketoE2, AN, 17OHP5 and 21-deoxyF were never detected (Table 1). Although the applicability of the present method for these five non-detected hormones cannot be demonstrated, these results suggest that better sensitivity is necessary to detect these hormones in hair. This might be helpful to researchers in the design of future methods targeting hormones for which no information is available, and particularly on the concentration range to be expected in a biological matrix. Several hypotheses may be proposed to explain the nondetection of some compounds, such as the insufficient sensitivity of this method for these chemicals, which is a common drawback associated with multi-analyte methods. The hair length analyzed here (12 cm) may also have lowered the concentrations of some compounds due to washout or degradation, as previous studies have reported decreasing concentration of some hormones (e.g. E, F (15), 17OHP5, and 4-MeOE1 (16)) from proximal to more distal successive hair segments, though not others such as DHEA and P4 (15). One of the main advantages of hair analysis lies in the possibility to cover extended periods of time, thus giving access to averaged values and avoiding bias due to short-term variability. We therefore selected a 12-cm length to cover an entire year and limit the bias due to seasonal variability that was reported for some hormones (e.g. T, DHEA, P4, E, and F) (17). The analysis of long hair segments might however be associated with the possible drawback of concentration decrease over time, which should be taken into account in results interpretation. It is thus necessary to consider hair length when comparing hormone concentration between different studies, and also to normalize sample length in studies investigating hair hormones in relation to biological outcomes or external stressors. The homogeneity of our study population (only women with a narrow age range) can also explain the low detection frequency and concentrations observed here for some compounds, which would present at higher levels in other individuals, such as testosterone in males or estriol in pregnant women. Similarly, other compounds could also present at higher levels in individuals with endocrine-related diseases. For example, elevation in serum 11-deoxycortisol (11-deoxyF) and 11-deoxycorticosterone (11-DOC) levels have been demonstrated in patients with 11β-hydroxylase deficiency (18, 19). They are thus very likely to be detectable in such patients’ hair, given their respective detection rates of 19 and 87% in our healthy study population. The fact that these compounds were not detected in 100% of the subjects under study should therefore still allow using this method in such clinical context.

In our study population, complete reference intervals were established for E1, E2, AD, DHEA, P4, 17OHP4, E, F, and T3, with DHEA, P4 and E showing the highest concentrations. Consistent with our results, previous studies have demonstrated higher hair concentrations of DHEA relative to T (20) and E relative to F (21, 22). Data on reference intervals are lacking for these hormones in human hair, except for E and F. In a population-based Dutch cohort with 295 adults, reference ranges of 3.29–20.48 and 0.68–10.49 pg/mg were found for hair E and F (23), which are comparable to those observed in our study population (5.08–37.3 and 1.91–11.0 pg/mg, respectively), though different length of hair segments (0–3 cm vs 0–12 cm) were used between the two studies.

Our results confirm the strong positive correlation between hair E and F concentrations found in a large occupational cohort (*n*  = 1258) (21) and a small cohort (*n*  = 62) (24). The significant positive correlations observed between precursors and metabolites (e.g., AD/T, DHEA/AD, 17OHP4/AD, P4/17OHP4, E/F, E/THE, F/THF, T3/T2, and T4/T3) demonstrate the reliability of the results obtained from our method and the applicability of hair analysis to hormonal assessment. These correlations may help to highlight altered metabolic pathways of hormones, although they need to be confirmed in other populations because this is the first study to reveal correlations between multiple hormones in human hair.

We observed the inverse correlations documented in plasma and/or serum between age and concentrations of AD, T, DHEA and DHEAS for premenopausal women (25, 26) or between age and T4 levels (27), despite the narrow age range (25–45 years) of our study population. Moreover, the positive correlation of hair E and F concentrations with age and BMI agree with the results of a large German study (21). Likewise, metabolites of E and F (i.e. THE, THF and αTHF) were also positively correlated with BMI. These results suggest that hair analysis could help to identify endocrinological disorders or to observe the effects of external stressors on hormonal status/metabolism (e.g. exposure to endocrine disruptors).

In summary, we developed a method for analyzing a panel of 37 endogenous hormones and metabolites in human hair. Applying this method to a middle-aged female population enabled to detect 32 compounds in at least one subject, and 9 were detected at percentile 2.5 or below. This method needs to be applied to different population groups and in different contexts (e.g. clinical settings, populations exposed to endocrine disruptors…) for correctly appreciating its potential to reveal hormonal disorders, especially regarding the compounds not detected in all samples.

## Supplementary Material

Supplementary Materials

Table S1. Literature review on endogenous hormones measured in aldut hair.

Table S2. Method validation results.

Table S3. Conditions of liquid chromatography-tandem mass spectrometry.

Table S4. Hormones concentrations (pg/mg) in the pooled hair used as matrix for method validation and sample analysis

Supplementary Figure 1

Supplementary Figure 2

Supplementary Figure 3

Supplementary Figure 4

Supplementary Figure 5

## Declaration of interest

The authors declare that there is no conflict of interest that could be perceived as prejudicing the impartiality of this article.

## Funding

Financial support from L’Oréal for the hair analysis performed on the Chinese women.
