# Supplementary material for: Profiling steroid and thyroid hormones with hair analysis in a cohort of women aged 25 to 45 years old
Source: Eur J Endocrinol. 2022 Feb 21;186(5):K9–K15. doi: 10.1530/EJE-22-0081 (PMC8942333; doi:10.1530/EJE-22-0081)
Supplement: Supplementary Materials [file supplementary_material.pdf]

Supplementary materials and methods

Chemicals and reagents

Endogenous hormones analyzed in this study consisted of 11 estrogens, 7 androgens, 4 progestogens, 10 corticosteroids, 4 thyroid hormones, and 1 pineal hormone (Fig. 1). Estrone (E1), androstenedione (AD), 5α-dihydrotestosterone (DHT), dehydroepiandrosterone (DHEA), androsterone (AN), progesterone (P4), 17α-hydroxyprogesterone (17OHP4), 11-deoxycorticosterone (11-DOC), corticosterone (B), cortisol (F) together with internal standard (ISTD) testosterone-d3 (T-d3) were purchased from LGC Standards (Molsheim, France). 17β-estradiol (E2), estriol (E3), T, pregnenolone (P5), cortisone (E), 3,3',5-triiodo-L-thyronine (T3), 3,5,3',5'-triiodo-L-thyronine (T4), melatonin (MEL) together with ISTDs AD-13C3, DHEA-d6, P4-d9, 21-deoxycortisol-d8 (21-deoxyF-d8), F-d4, tetrahydrocortisol-d5 (THF-d5), 3,3'-diiodo-L-thyronine-13C6 (T2-13C6), T3-13C6, 3,3',5'-triiodo-L-thyronine-13C6 (rT3-13C6), T4-13C6 and MEL-d4 were obtained from Sigma Aldrich (Overijse, Belgium). Epitestosterone (EpiT), dehydroepiandrosterone sulfate (DHEAS), 2-hydroxyestrone (2-OHE1), 4-hydroxyestrone (4-OHE1), 16α-hydroxyestrone (16-OHE1), 2-methoxyestrone (2-MeOE1), 4-methoxyestrone (4-MeOE1), 2-methoxyestradiol (2-MeOE2), 4-methoxyestradiol (4-MeOE2), 16-ketoestradiol (16-ketoE2), 17α-hydroxypregnenolone (17OHP5), aldosterone (ALD), 11-deoxycortisol (11-deoxyF), 21-deoxyF, tetrahydrocortisone (THE), THF, 5α-tetrahydrocortisol (αTHF), T2, rT3 together with ISTDs E1-d4, E2-d4, E3-d3, 2-OHE1-d4, 4-MeOE2-d3, 16-ketoE2-d5, ALD-d7, E-d8 and B-d8 were purchased from Toronto Research Chemicals (Toronto, Canada). All standards and internal standards had purities ≥ 95%, except for T-d3 and T3-13C6 (91% and 89.6%, respectively).

Analytical grade acetone and methanol (MeOH) along with ULC/MS grade acetonitrile (ACN), MeOH, water and formic acid were supplied by Biosolve (Valkenswaard, The Netherlands). Ultrapure Milli-Q water was obtained from an AFS-8 system (Millipore, Brussels, Belgium). Ammonia solution (NH3, 25%) was purchased from Merck (Darmstadt, Germany). Sodium dodecyl sulfate (SDS), sodium bicarbonate (NaHCO3) and derivative agent dansyl chloride were purchased from Sigma Aldrich (Overijse, Belgium), and sodium hydroxide (NaOH) from VWR Chemicals (Darmstadt, Germany). Bond Elut C18 cartridges (50 mg, 1 mL) were bought from Agilent technologies (Santa Clara, CA, USA).

Preparation of stock and working standards

Individual stock solutions of melatonin (MEL) and steroid hormones were prepared at concentrations of 1 or 0.5 mg/mL in acetonitrile (ACN) or methanol (MeOH) while those of thyroid hormones were prepared at 0.1 mg/mL concentration in MeOH with 0.1 M ammonia solution (NH_3). Individual stock internal standard (ISTD) solutions were prepared at concentration of 0.1 mg/mL in ACN,

MeOH or MeOH with 0.1 M NH_3. Working standard solutions were prepared in MeOH at final concentrations of 0.5, 1, 2.5, 5, 10, 25, 50, 100, 250, 500 and 1000 ng/mL. A working ISTD solution containing all the 21 ISTDs was prepared in MeOH at 100 ng/mL concentration, except for aldosterone-d7 (ALD-d7; 1000 ng/mL). All stock and working solutions were stored in amber glass bottles at -20 °C.

Sample collection

Hair samples were collected in 2016 from 204 healthy Chinese women aged between 25 and 45 years. Details on the population, recruitment, and sampling procedures have been previously reported (1, 2, 3). Briefly, the study population were non-smokers and non-alcoholics with natural hair from root to tip with a length ≥ 30 cm. Hair strands were cut scalp-near with scissors from the posterior vertex region, wrapped in aluminium foils, and stored in kraft envelopes at room temperature. The first 12-cm hair segment from the root was used to analyze hormones, which represents the average hormone levels over a 12-month period prior to hair sampling by assuming an average hair growth rate of 1 cm/month (4). Only 196 samples were included in the present study because the sample amount was insufficient for eight subjects after analysis of other measurements (1, 2, 3). Written consent has been obtained from each subject after full explanation of the purpose and nature of all procedures used. The study was approved by the Ethics Committee of the Chinese Academy of Inspection and Quarantine Cosmetics Tech Center (protocol n°: 2015-033-DY-024) (1).

Sample preparation

Hair samples were washed by shaking them in 5% sodium dodecyl sulfate (SDS) in water for 5 min, followed by Milli-Q water for 1 min and MeOH for 5 min at room temperature (5). After decontamination, hair strand was placed in a fume hood to dry for 2 h and pulverized in a ball mill. 50 ± 0.05 mg of hair powder was weighed into a 5-mL glass tube, added with 10 µL of working ISTD solution and 2 mL of MeOH, and incubated for 24 h at 40 °C with gentle shaking for hormones extraction. After centrifugation (10 min, 4000 g, room temperature), 1.6 mL of clear supernatant was transferred into a new glass tube and evaporated to dryness at 40 °C under a gentle stream of nitrogen. The dry residue was resuspended in 1 mL of 5% MeOH in water and subsequently purified by solid-phase extraction (SPE) using a Bond Elut C_{18} cartridge preconditioned with 1 mL of MeOH followed by 1 mL of Milli-Q water. After loading the sample, the SPE cartridge was washed with 1 mL of 10% MeOH in water, vacuum dried for 30 min, and eluted three times with 0.7 mL of MeOH each. After evaporation at 40 °C under nitrogen, the dry residue was reconstituted in 160 µL of 50% MeOH in water, and split for analysis of estrogens (60 µL) with dansyl chloride derivatization (6) and analysis of the remaining hormones without

derivatization. Extracts were centrifuged (10 min, 20000 g, 4 °C) and transferred to injection vials for LC-MS/MS analysis.

Liquid chromatography-tandem mass spectrometry (LC-MS/MS) method

Hormones were measured using a Waters Acquity UPLC H-class system (Wexford, Ireland) coupled to Waters Xevo TQ-S triple quadrupole mass spectrometer (Wexford, Ireland) with an electrospray ionization source (ESI). The chromatographic separation was achieved on a Waters Acquity BEH C_{18} column (100×2.1 mm, 1.7 μm particle size) protected by a frit filter of 0.2 μm (Wexford, Ireland). Autosampler and column temperature were set at 10 and 40 °C, respectively. The mobile phase A was 0.1% formic acid in water, and B was MeOH. The flow rate was 0.35 mL/min. Two different gradient programs were developed for the hormones with (i.e., estrogens; group 1) and without (i.e., the remaining hormones; group 2) derivatization, as they had to be injected separately and as there were isobaric analyte pairs in both groups (Table S3). The injection volume of both groups were 10 μL. The corresponding total analysis time per injection were respectively 20 and 18 min, including column re-equilibration (5 min each).

Mass spectral data were acquired in multiple reaction monitoring (MRM) mode with two transitions per analyte and one per ISTD. Optimized MRM transitions, cone voltages and collision energies are shown in Table S3. Positive ionization mode was employed for all hormones, with the exception of dehydroepiandrosterone sulfate (DHEAS), tetrahydrocortisone (THE), tetrahydrocortisol (THF) and 5α-tetrahydrocortisol (αTHF). Optimized MS parameters were: source temperature at 150 °C, capillary voltage at 2.8 kV, source offset at 60 V, desolvation gas (N_2) at 1200 L/h and 650 °C, cone gas (N_2) flow at 150 L/h, collision gas (argon) flow at 0.15 mL/min, and nebulizer gas (N_2) at 6.0 bar. Data acquisition, data processing and peak integration were conducted using the MassLynx 4.2 software.

Method validation

The method validation was performed in quintuplicate using human hair spiked with all target hormones by determining linearity, lower limit of quantification (LLoQ), accuracy, precision, recovery, selectivity and carry-over. Here, assessing matrix effect was not necessary because the calibration curve was built with supplemented hair samples. To do this, a pool of hair collected from three healthy individuals was prepared to serve as hair matrix. Hormones levels in the pooled hair are presented in Table S4. The pooled human hair was analyzed in the same way as real samples but supplemented with 10 μL of appropriate working standard solution to obtain concentrations of 0, 0.1, 0.2, 0.5, 1, 2, 5, 10, 20, 50, 100 and 200 pg/mg for calibration curve construction.

Our acceptance criteria for linearity were correlation coefficient (r^2) ≥ 0.99 and each calibrator with a percent relative error (%RE) ≤ 25% and an imprecision (%coefficient of variation (CV)) ≤ 25%. LLoQ was defined as the lowest calibrator of each analyte that met the validation criteria. Intra- (quintuplicates within one batch) and inter-assay (one replicate over five batches) accuracy (%RE) and precision (%CV) were assessed for each of the calibrators. Recovery was assessed at three calibrators (1, 10 and 100 pg/mg) in quintuplicate, and was calculated as: R (%) = (A-B) / (C-B) × 100, where A is the analyte peak area in the pooled hair sample spiked with standards before extraction; B is that in the pooled hair sample without spiking standards; and C is that in the pooled hair sample spiked with standards just before injection. Selectivity was confirmed by the ratio between the qualitative ion and quantitative ion with variability within 25%. Carryover was checked by injecting one blank solvent (MeOH) following the highest calibrator (100 pg/mg for estrogens and 200 pg/mg for the remaining hormones) and comparing the peak area in MeOH with that in the lowest calibrator. The acceptance criterion was carry-over below 20% for standards and below 5% for ISTDs.

Statistical analysis

Data analysis was performed using R software with R-package "NADA" (version 3.6.1). Because hormone concentrations did not follow normal distribution and there were nondetects (i.e., censored data) for some hormones, the Kaplan-Meier method (7) was used to estimate descriptive statistics including 2.5th, 25th, 50th, 75th, and 97.5th percentiles. Kendall rank correlation was conducted using the cenken function to evaluate correlations between hormones, age, and body mass index (BMI). This analysis was limited to hormones detected in > 50% of hair samples to avoid bias caused by censored data. Kendall's τ coefficients ($\tau_{Kendall}$) are presented in a heatmap. Significance level was set at $p < 0.05$.

References

1. Peng F-J, Hardy EM, Mezzache S, Bourokba N, Palazzi P, Stojiljkovic N, Bastien P, Li J, Soeur J & Appenzeller BMR. Exposure to multiclass pesticides among female adult population in two Chinese cities revealed by hair analysis. *Environment International* 2020 **138** 105633.
2. Palazzi P, Mezzache S, Bourokba N, Hardy EM, Schritz A, Bastien P, Emond C, Li J, Soeur J & Appenzeller BMR. Exposure to polycyclic aromatic hydrocarbons in women living in the Chinese cities of BaoDing and Dalian revealed by hair analysis. *Environment International* 2018 **121** 1341-1354.
3. Peng F-J, Hardy EM, Béranger R, Mezzache S, Bourokba N, Bastien P, Li J, Zaros C, Chevrier C, Palazzi P *et al*. Human exposure to PCBs, PBDEs and bisphenols revealed by hair analysis: A comparison between two adult female populations in China and France. *Environmental Pollution* 2020 **267** 115425.
4. Kintz P, Salomone A & Vincenti M. Hair analysis in clinical and forensic toxicology (first ed.). Academic Press, Elsevier, Amsterdam, 2015.
5. Grova N, Wang X, Hardy EM, Palazzi P, Chata C & Appenzeller BMR. Ultra performance liquid chromatography – tandem mass spectrometer method applied to the analysis of both thyroid and steroid hormones in human hair. *Journal of Chromatography A* 2020 **1612** 460648.
6. Xu X, Keefer LK, Ziegler RG & Veenstra TD. A liquid chromatography–mass spectrometry method for the quantitative analysis of urinary endogenous estrogen metabolites. *Nature Protocols* 2007 **2** 1350-1355.
7. Lee L. NADA: Nondetects and data analysis for environmental data. 2017 **1**.
