# Supplementary figures and images for: Profiling steroid and thyroid hormones with hair analysis in a cohort of women aged 25 to 45 years old
Source: Eur J Endocrinol. 2022 Feb 21;186(5):K9–K15. doi: 10.1530/EJE-22-0081 (PMC8942333; doi:10.1530/EJE-22-0081)

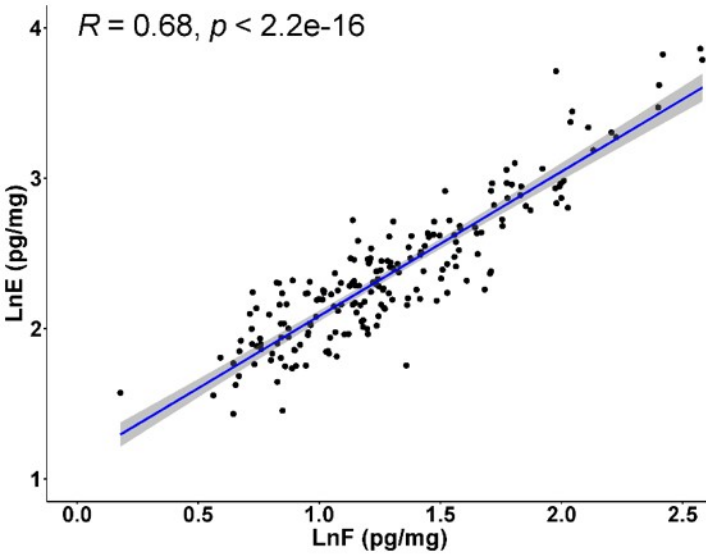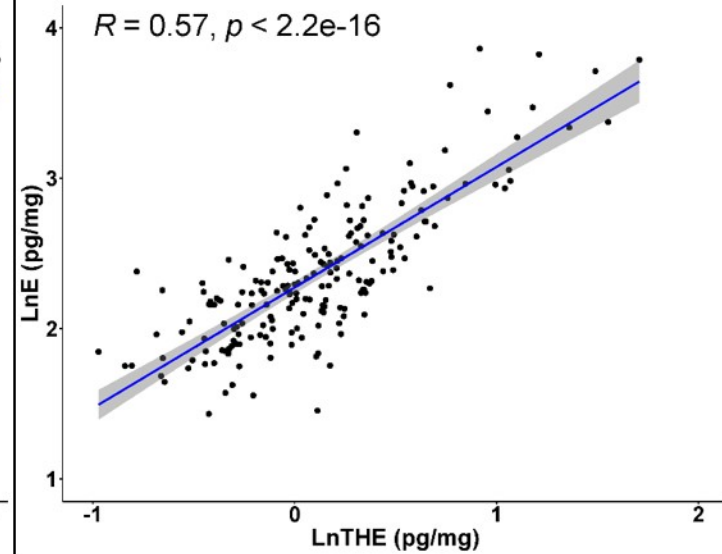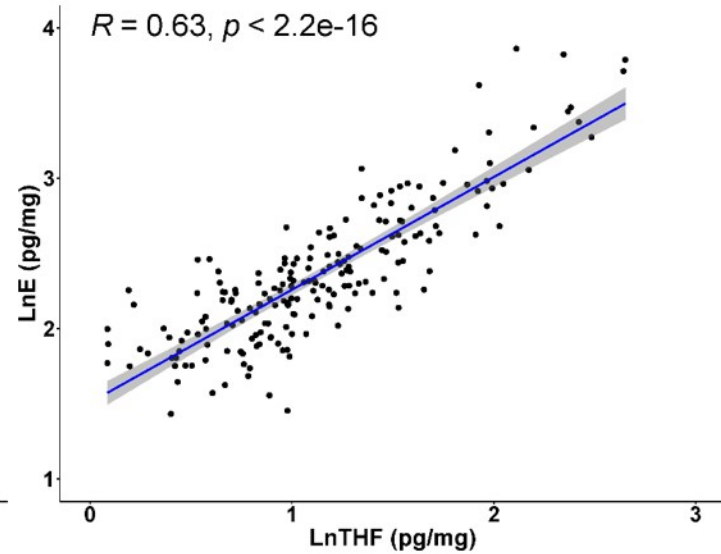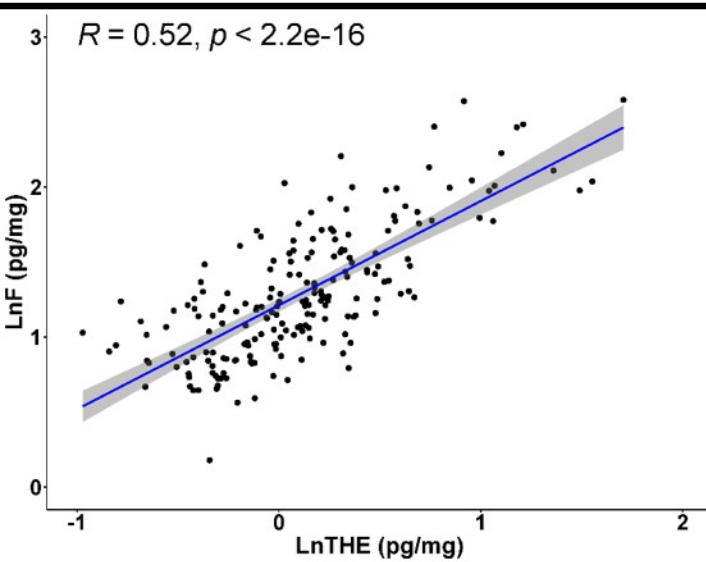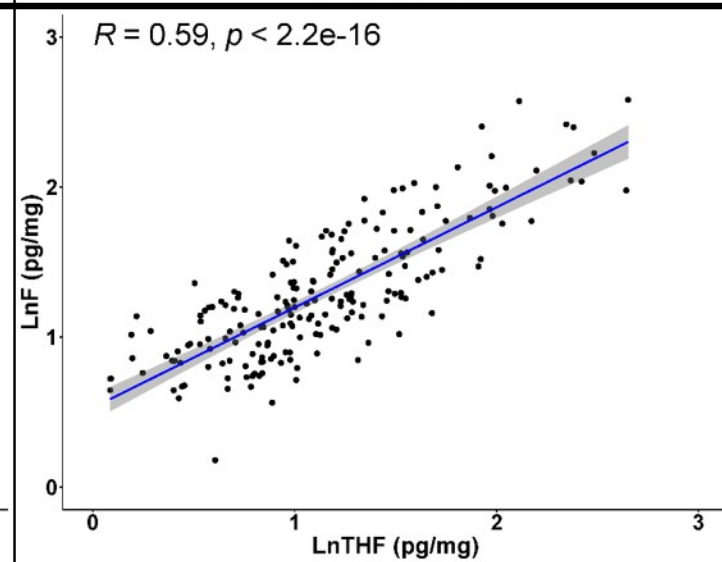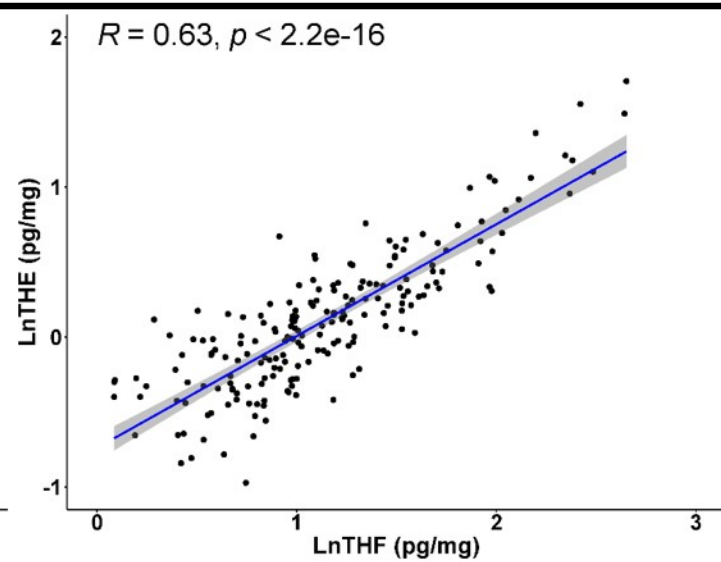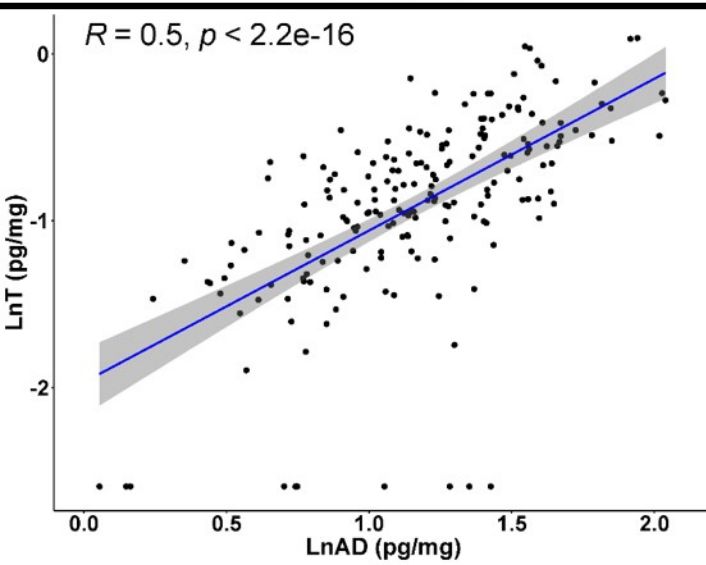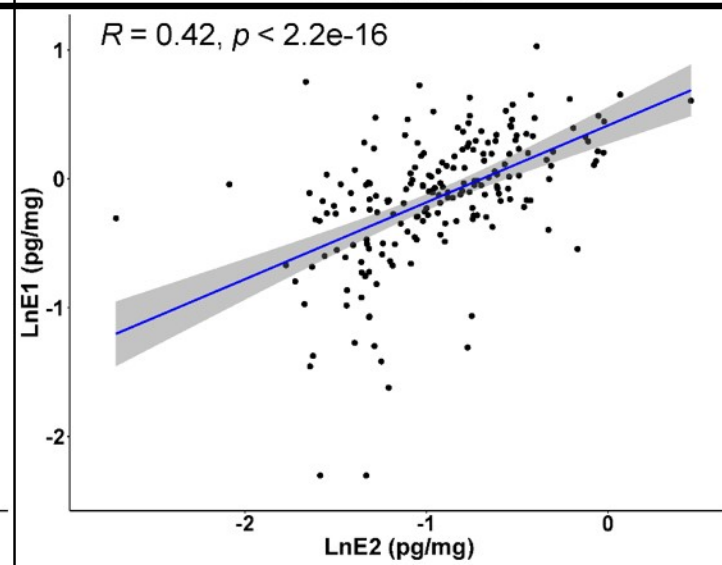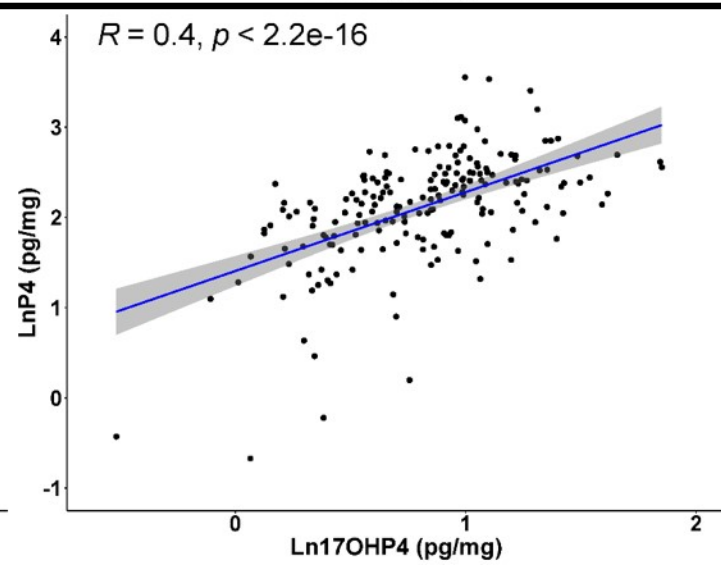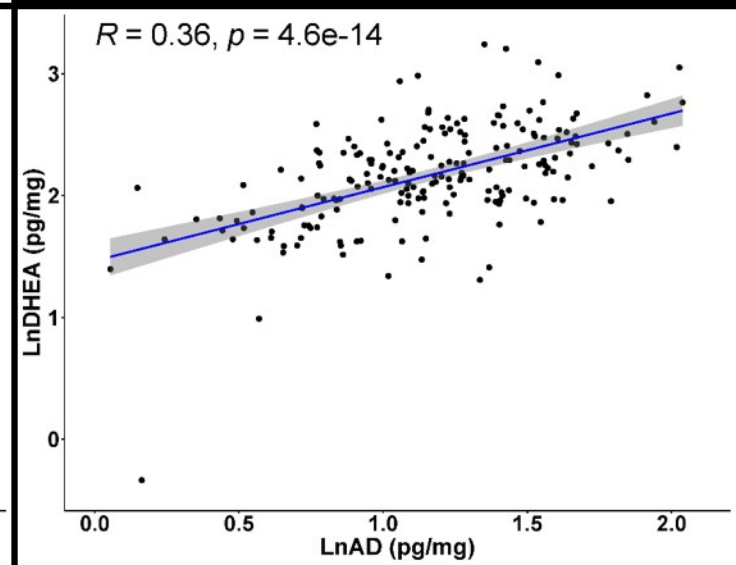

Supplement: Supplementary Figure 1 [file supplementary_figure_1.pdf]

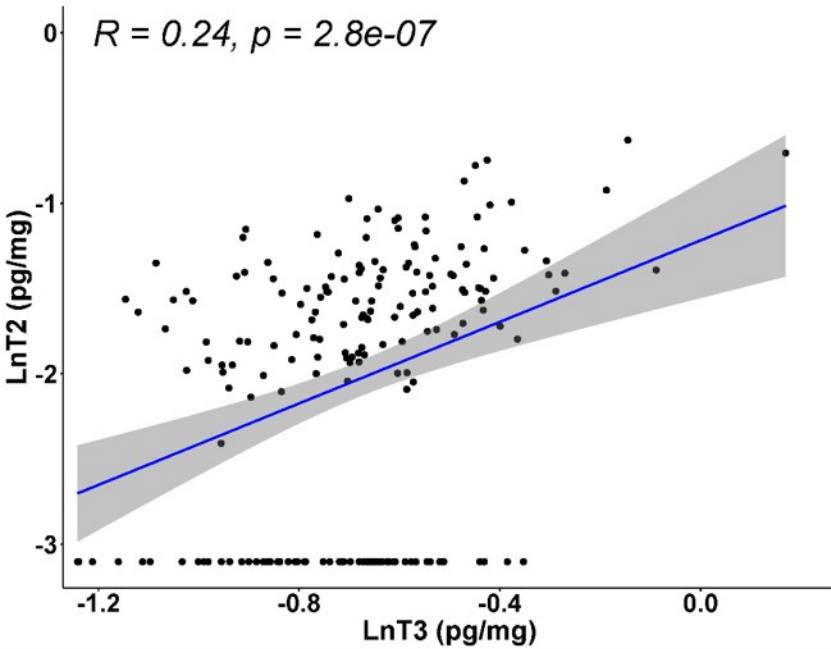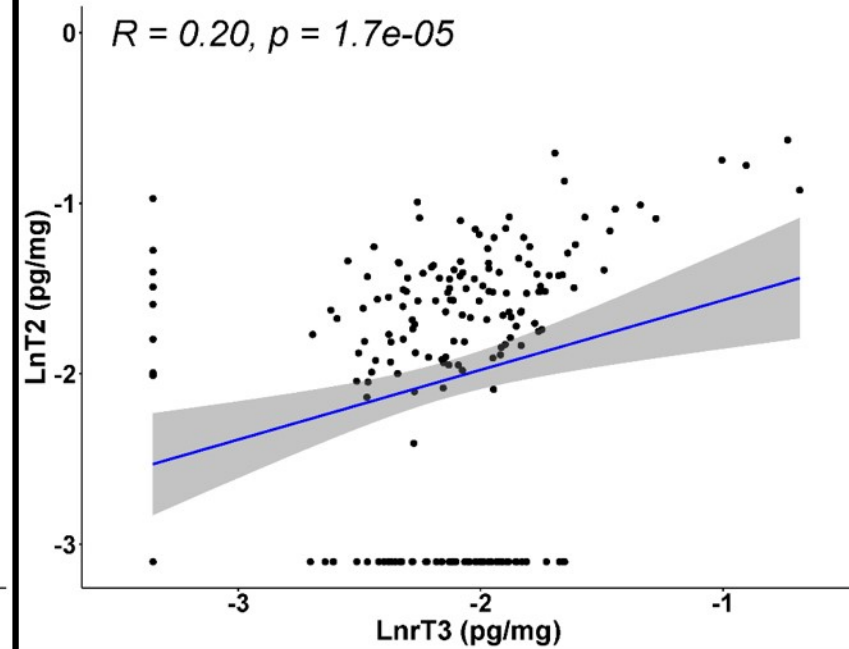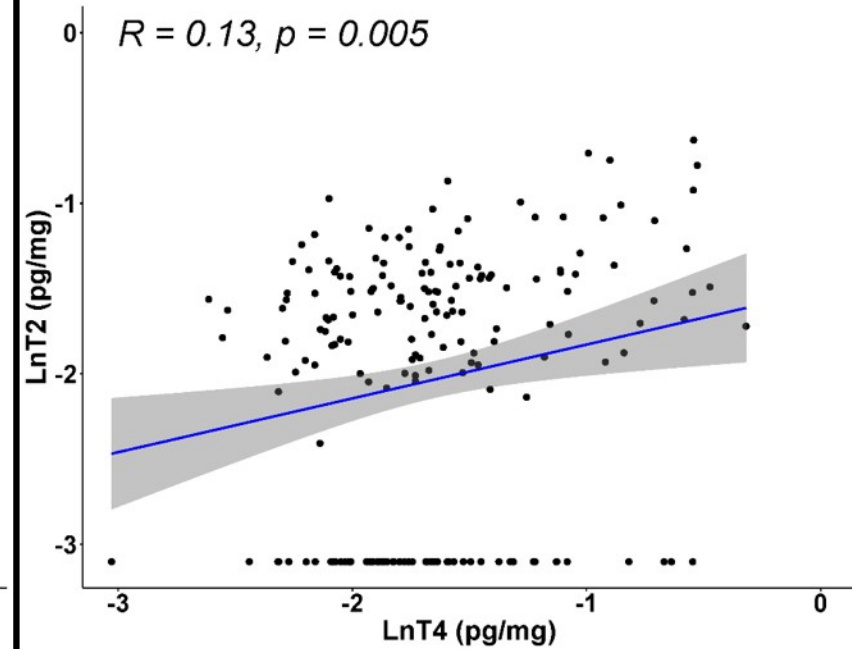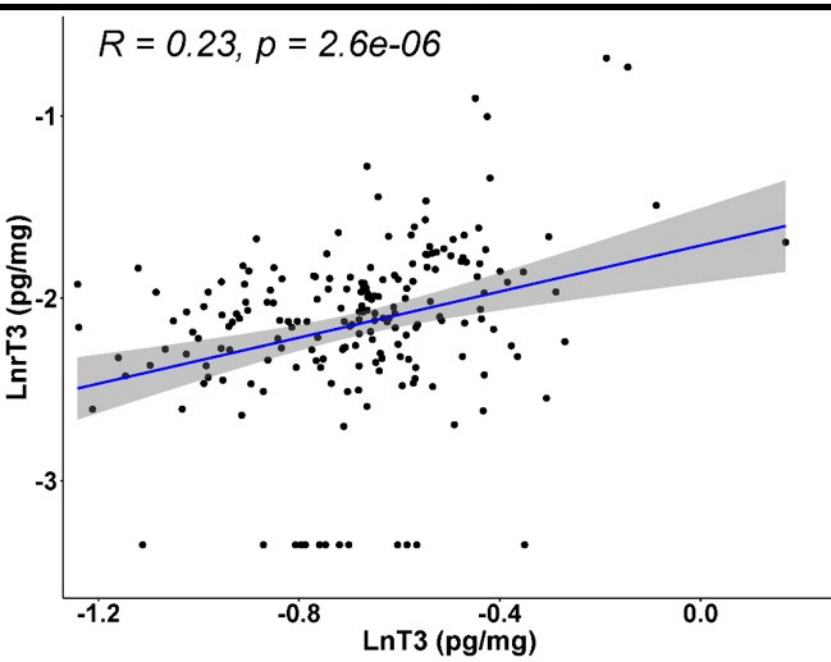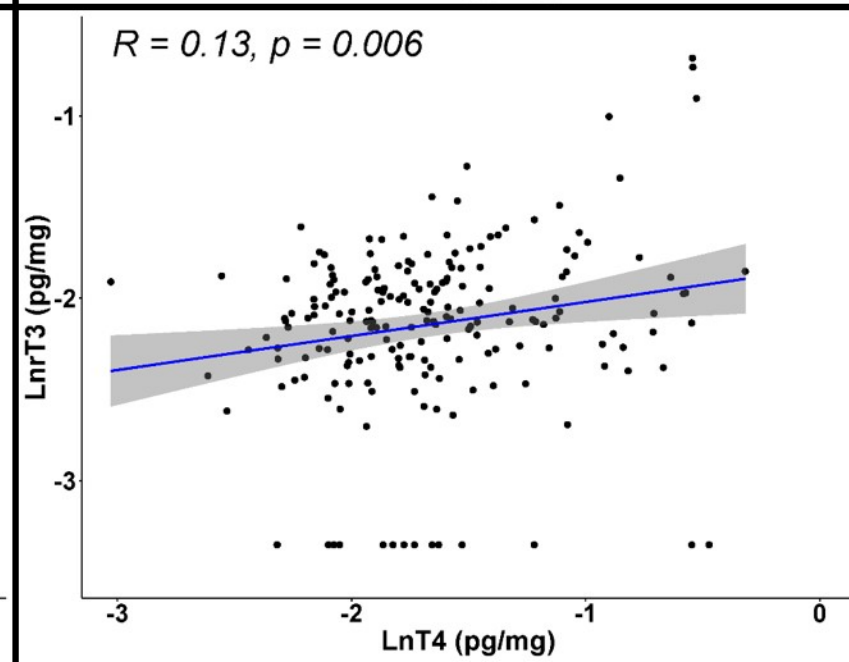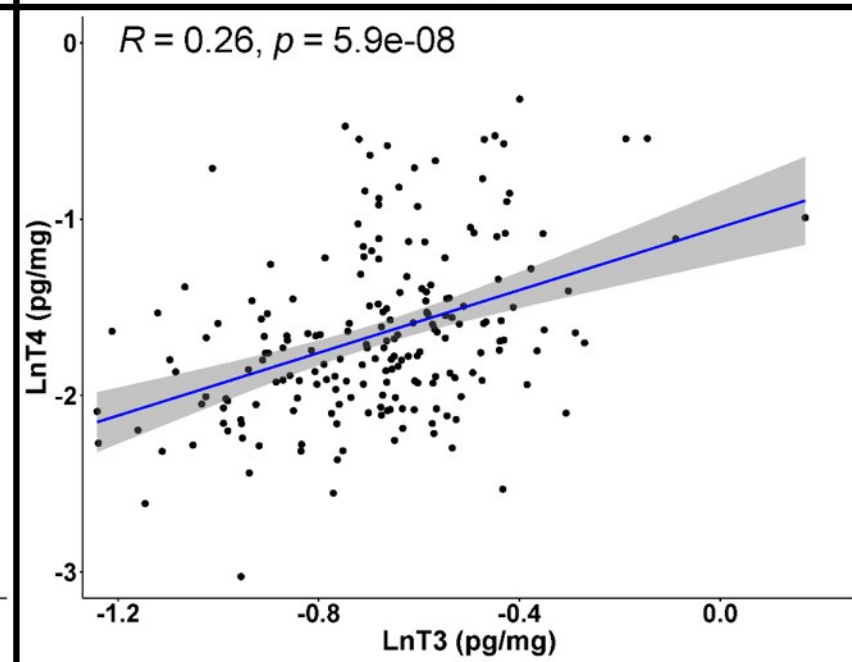

Supplement: Supplementary Figure 2 [file supplementary_figure_2.pdf]

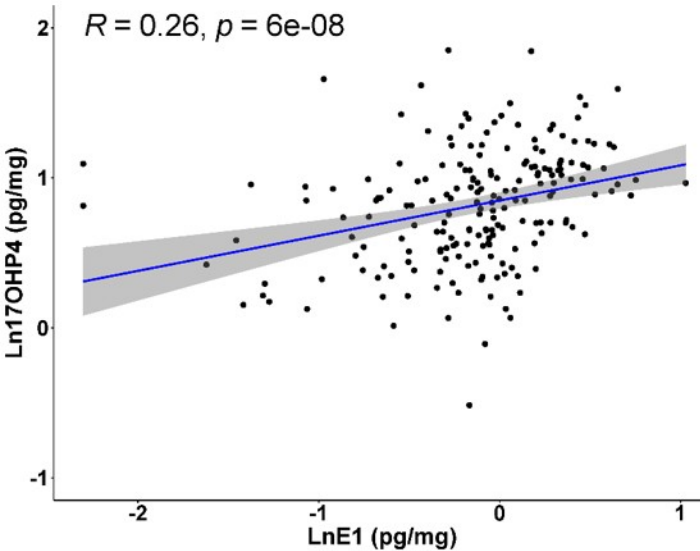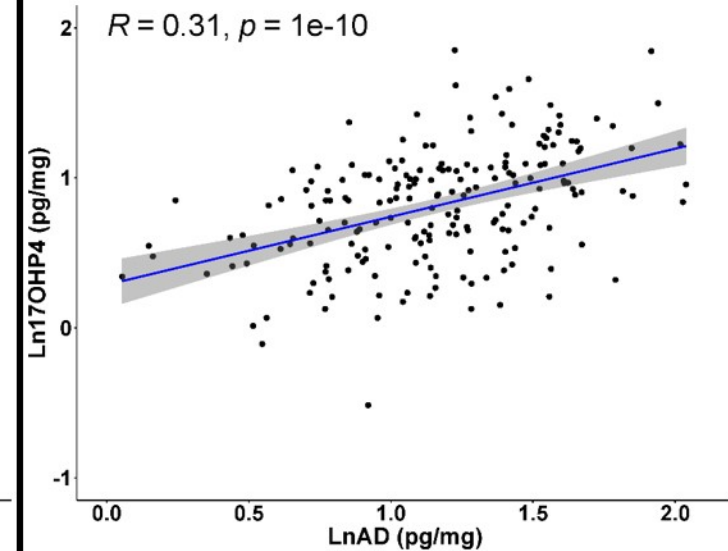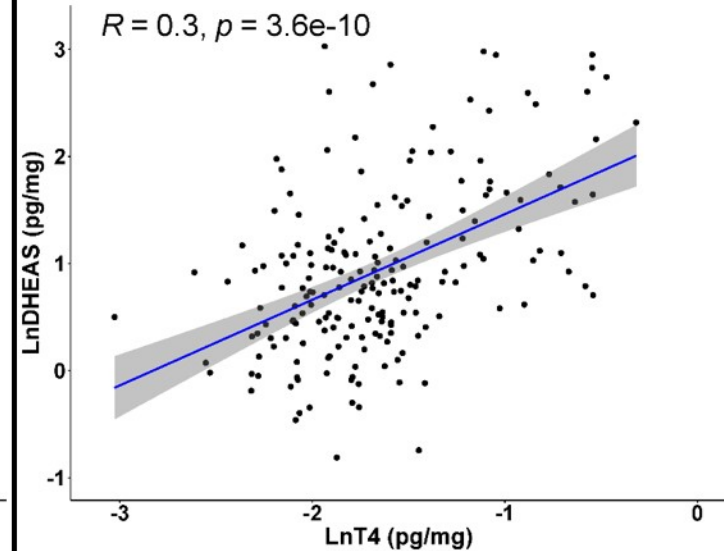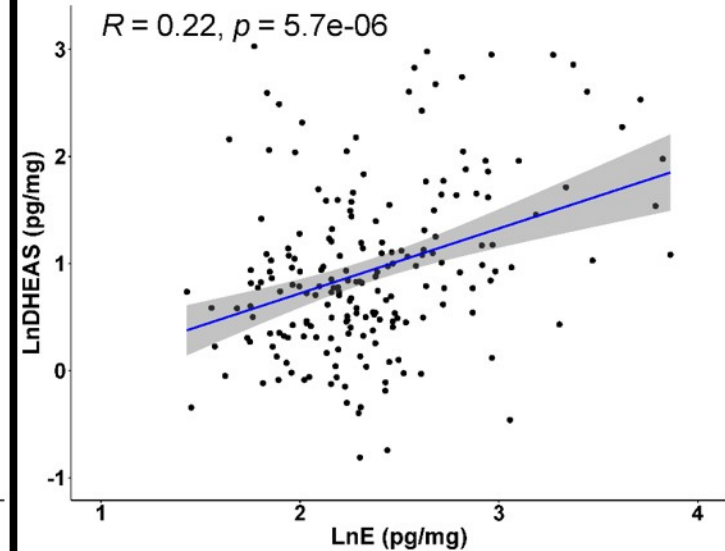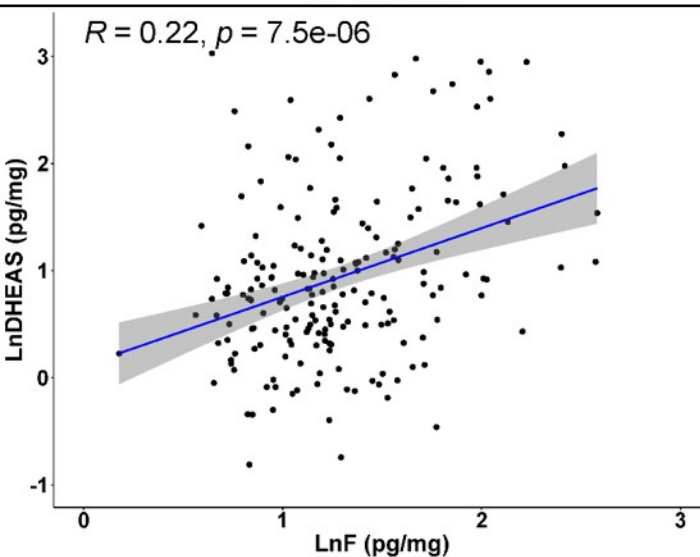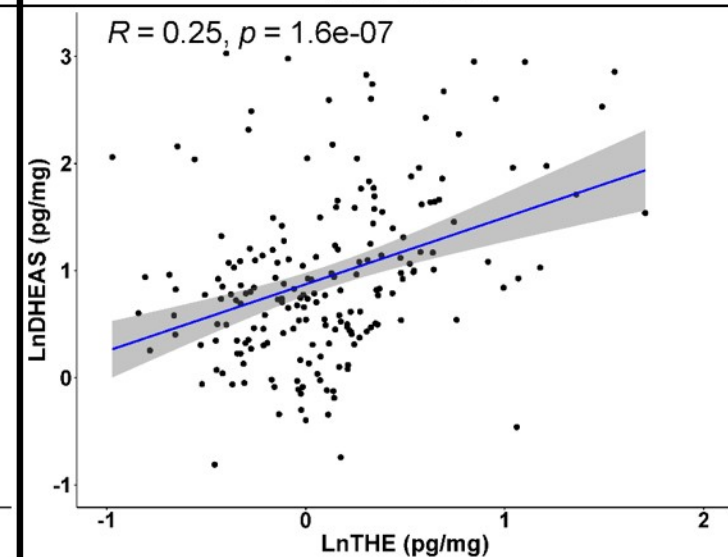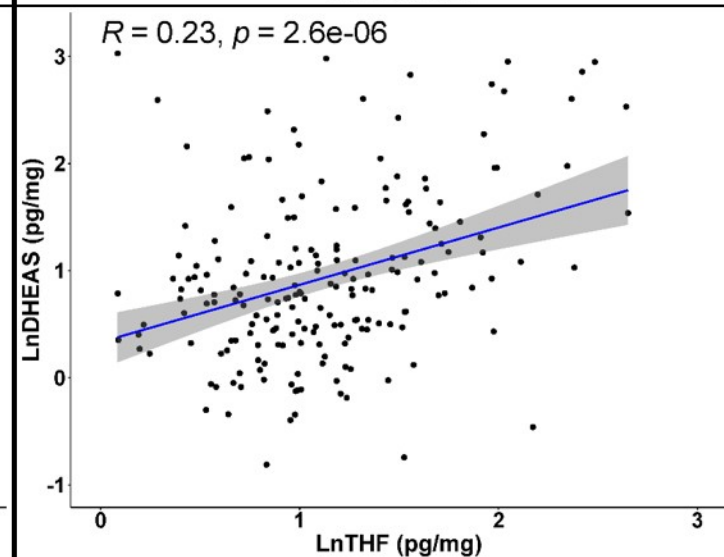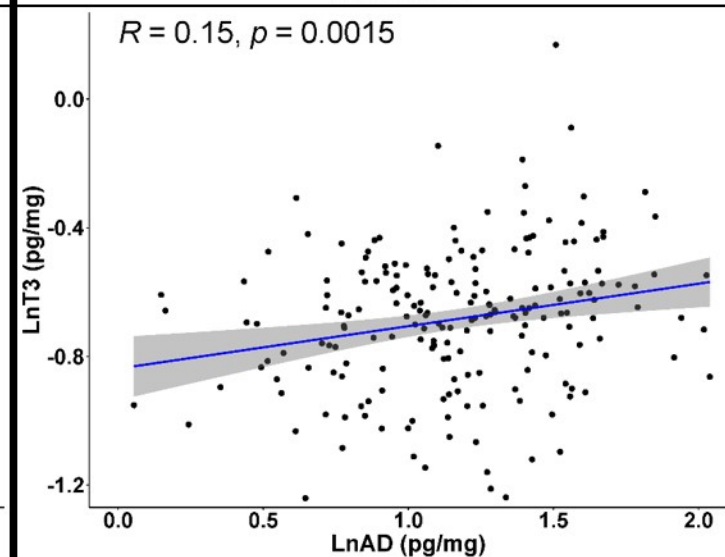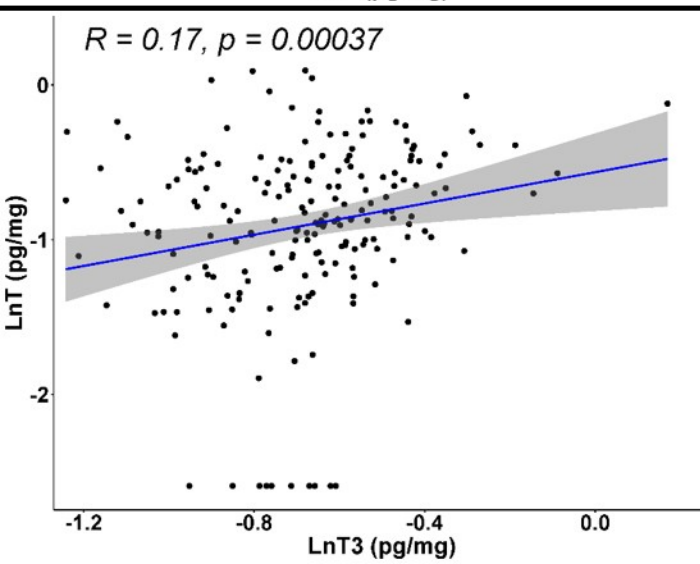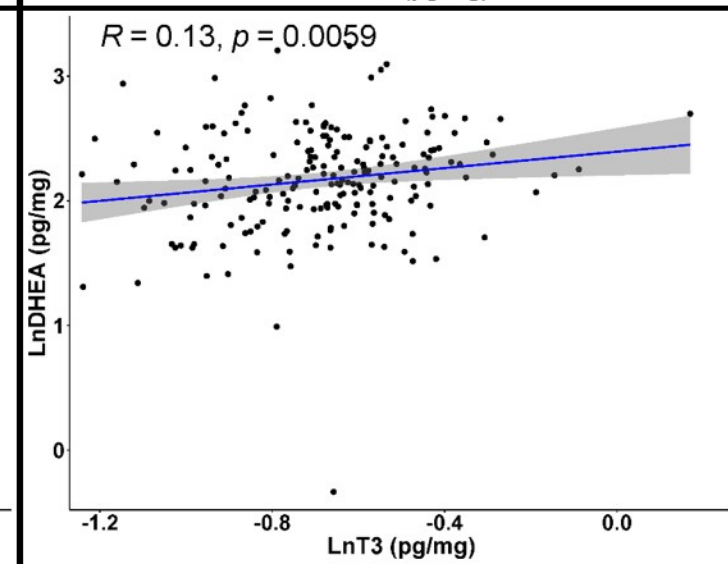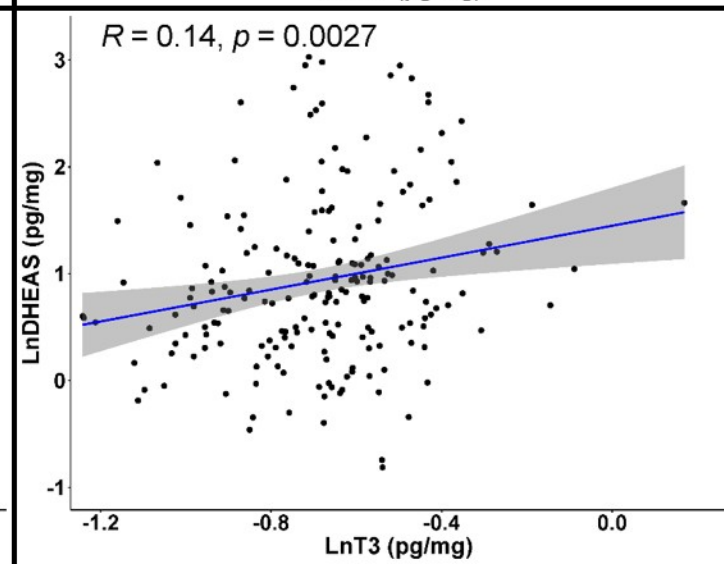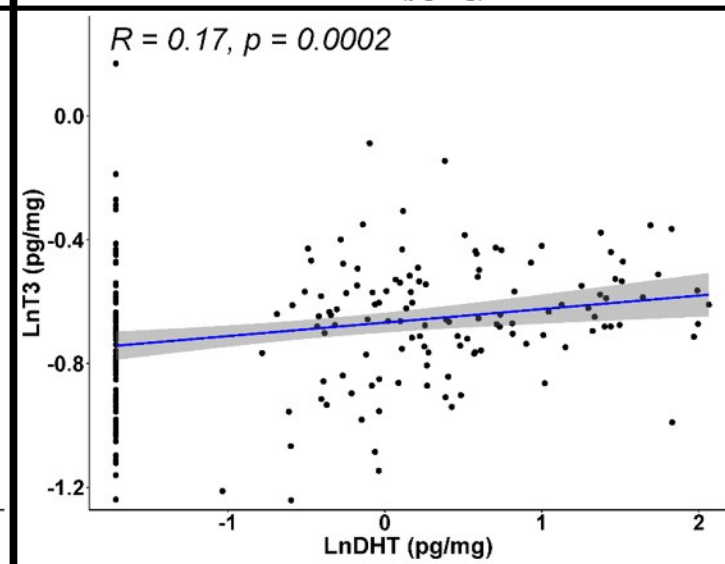

Supplement: Supplementary Figure 3 [file supplementary_figure_3.pdf]

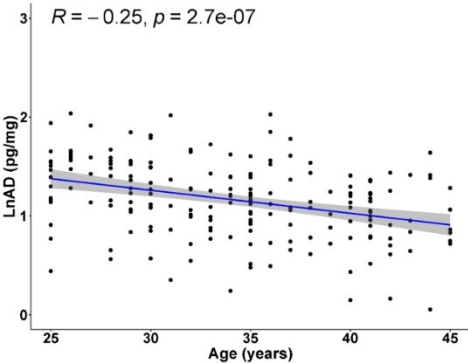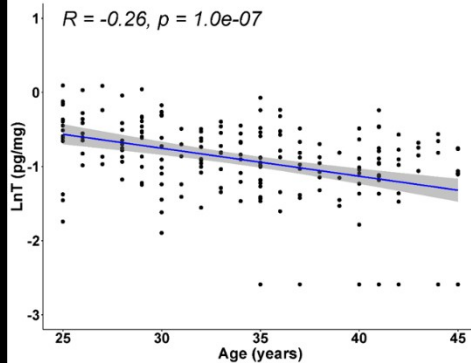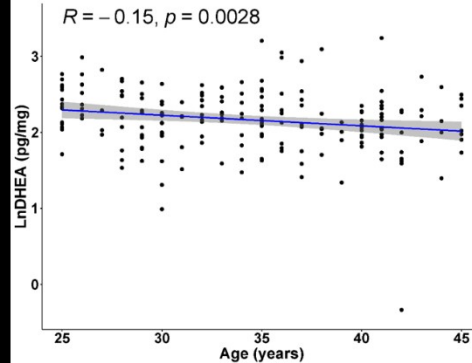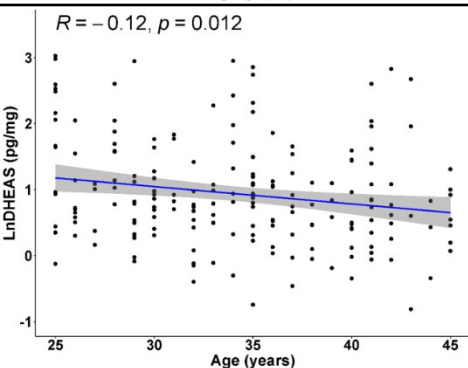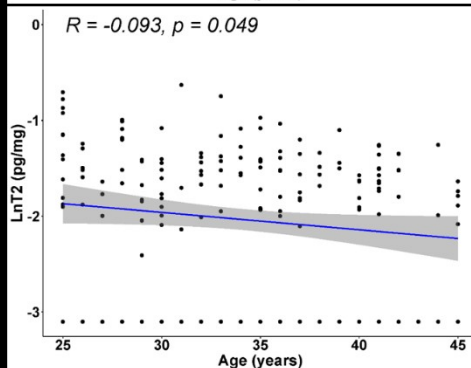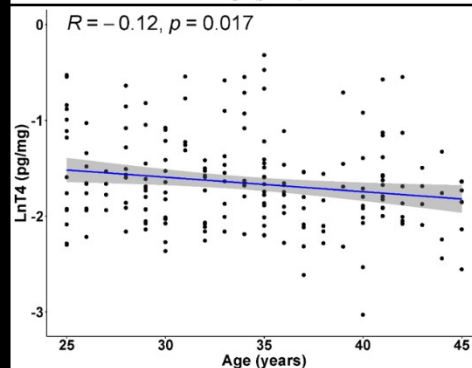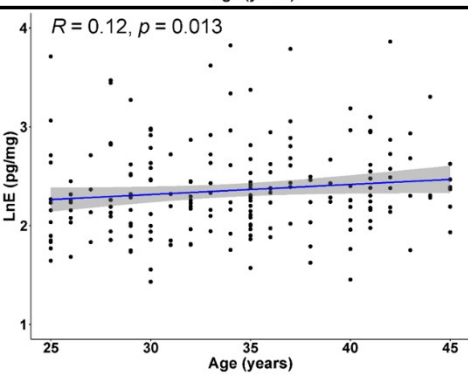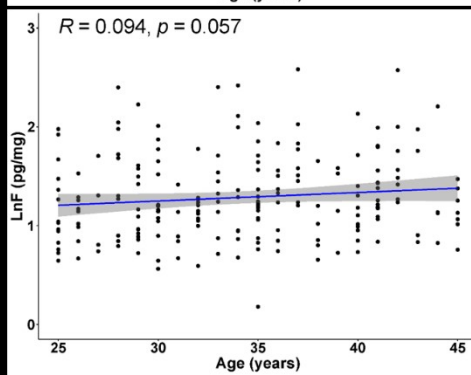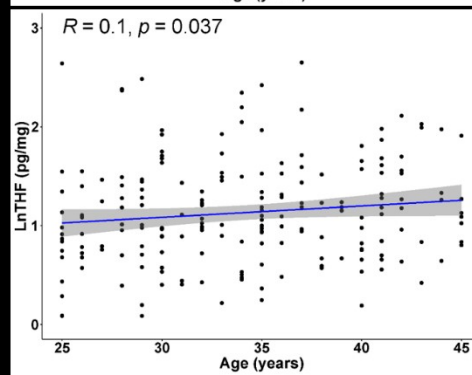

Supplement: Supplementary Figure 4 [file supplementary_figure_4.pdf]

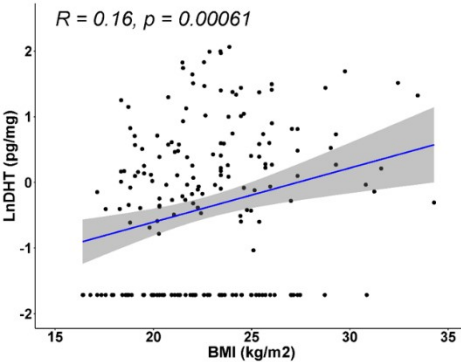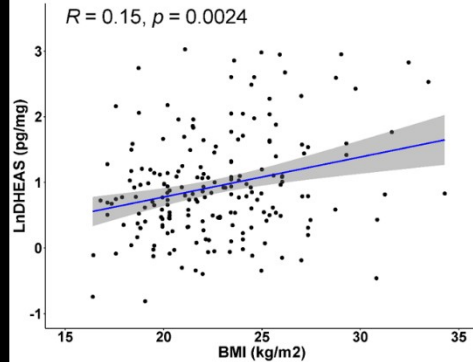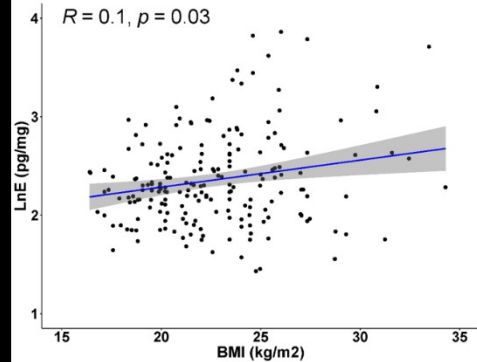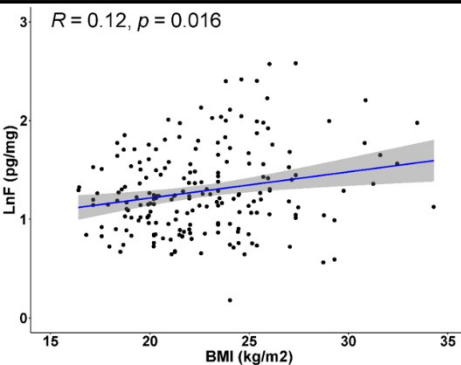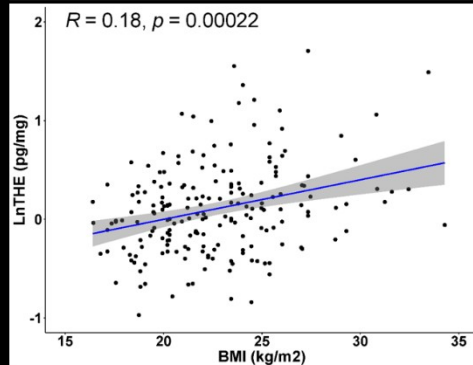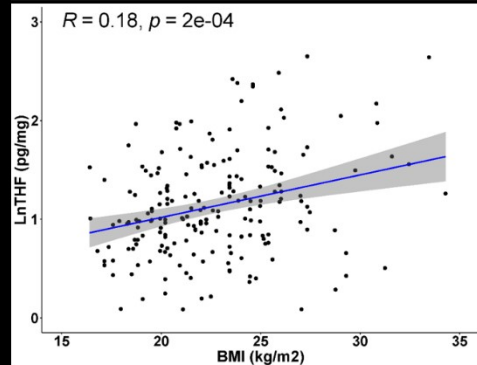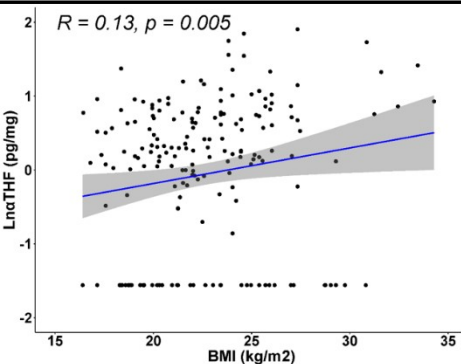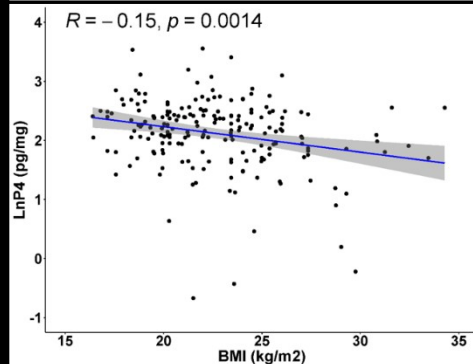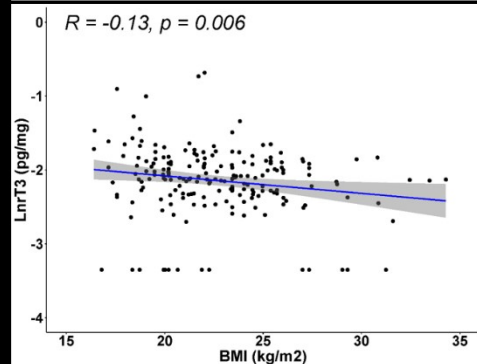

Supplement: Supplementary Figure 5 [file supplementary_figure_5.pdf]
